# αS-SETMAR: Inducing Protective Chaos in Glioblastoma?

**DOI:** 10.3390/cancers18132151

**Published:** 2026-07-03

**Authors:** Sarah-Anne David, Sara Benharrat, Oriane Lié, Ambre Dufresne, Jérôme Jaillet, Murielle Genty, Sylvaine Renault, Corinne Augé-Gouillou

**Affiliations:** 1 Université de Tours, INSERM, Imaging Brain & Neuropsychiatry, iBraiN U1253, 37032 Tours, France; s-a.david@hotmail.fr (S.-A.D.); sara.benharrat.pro@gmail.com (S.B.); lieoriane@gmail.com (O.L.); ambre.dufresne1@gmail.com (A.D.); jerome.jaillet@univ-tours.fr (J.J.); murielle.genty@univ-tours.fr (M.G.); 2 TISSIUM (Tissue Reconstructing Solutions), 74 Rue du Faubourg Saint-Antoine, 75012 Paris, France

**Keywords:** glioblastoma, SETMAR, chromosomal instability, cell cycle regulation, therapeutic potential

## Abstract

Background/Objectives: Glioblastoma remains the most aggressive and lethal form of brain cancer, with no effective cure to date. The molecular mechanisms sustaining its development and relentless proliferation are still not fully understood. SETMAR, a protein lysine methyltransferase involved in various DNA repair and chromatin processes, has been reported as dysregulated in several cancers, including glioblastoma. Interestingly, S-SETMAR, a shorter isoform of SETMAR, has been suggested to antagonize the oncogenic properties of the full-length protein. Here, we explored the cellular and molecular consequences of S-SETMAR overexpression in glioblastoma cells. Methods: We compared native glioblastoma cells (8MGBA) with a recombinant 8MGBA line stably over-expressing αS-SETMAR, a stable form of S-SETMAR, using complementary cellular and molecular approaches. Results: Overexpression of αS-SETMAR markedly prolonged the cell cycle duration (from 27 to 37 h), leading to a significant decrease in cell proliferation. Unexpectedly, αS-SETMAR triggered genomic alterations characterized by an increased DNA content and extensive chromosomal instability, including aneuploidy, chromoanasynthesis-like rearrangements, and tripolar mitoses. Moreover, αS-SETMAR-expressing cells displayed heightened sensitivity to stress conditions mimicking chemotherapy and radiotherapy, resulting in increased apoptosis. Conclusions: Our findings identify αS-SETMAR as a dual modulator of glioblastoma cell fate—simultaneously slowing proliferation and promoting chromosomal instability while enhancing vulnerability to genotoxic stress. These results suggest that αS-SETMAR could serve as both a prognostic marker and a potential therapeutic tool in glioblastoma management.

## Simple Summary

Glioblastoma is the most aggressive type of brain cancer, with very poor survival and limited treatment options. Understanding the mechanisms that control its growth is essential for developing new therapies. Here, we investigated the short form of SETMAR, αS-SETMAR, in glioblastoma cells. We found that elevating αS-SETMAR levels slowed cell division and reduced proliferation, while simultaneously inducing widespread chromosomal instability. These changes increased the cells’ sensitivity to stress, such as chemotherapy or radiotherapy, promoting cell death. Our findings reveal that αS-SETMAR has a dual role in regulating both glioblastoma cell survival and genome integrity, supporting its potential as a prognostic marker and as a target for therapeutic intervention.

## 1. Introduction

Glioblastoma (GB, World Health Organization grade IV gliomas) is the most common primitive malignant tumor of the central nervous system, and remains one of the deadliest human cancers [1]. Despite aggressive treatments, patients with GB have a 5-year survival rate of 5% and a median survival of only 15 months. In addition, GB displays a striking heterogeneity, both at the cellular and morphological scale [2]. Thus, the treatment of GB is still a big challenge, and this field is worth investigating. In a previous study, we demonstrated that S-SETMAR, when enriched in tissues surrounding GB, correlates with increased patient survival [3].

Originating 45 million years ago, *SETMAR* is a fusion gene only present in higher primates. It consists of three exons, the first two from the *SET* gene and coding for methyltransferase functions, and the third from the *Hsmar1* transposase gene and coding for recombinase functions. The full-length SETMAR protein (FL-SETMAR) is described as a genome keeper, expressed in main tissues, but with different levels. In cancer cells, the *SETMAR* gene is over-expressed, and FL-SETMAR sustains oncogenic processes, probably through its involvement in DNA repair by Non-Homologous End-Joining (NHEJ), replication stress response and chromosome decatenation (review in [4]). The role of FL-SETMAR in cell proliferation is more ambiguous since some studies show a positive correlation [5,6,7], while another suggests the contrary [8]. *SETMAR* pre-mRNA can undergo alternative splicing, leading to the production of shorter proteins. One of them, S-SETMAR, was first discovered in GB [9], and more recently in colorectal cancers [10]. In addition, mRNA coding for shorter variants has been detected in bladder cancer cells [11] and leukemias [12], but the corresponding proteins have not been investigated. S-SETMAR lacks a part of the pre-SET domain and the whole SET domain, both encoded by exon 2. As a result, S-SETMAR is unable to methylate proteins as FL-SETMAR does, albeit with a moderate efficiency [13,14]. On the other hand, it is less efficient than FL-SETMAR in promoting NHEJ and appears to be preponderant over FL-SETMAR in GB stem cells [9]. Finally, both proteins have retained the ability to bind to *Hsmar1* Terminal Inverted Repeats (TIRs).

Little is known about S-SETMAR mechanisms of action, and only limited information is available regarding the mechanisms governing its production, including exon 2 exclusion during pre-mRNA maturation [15]. Interestingly, S-SETMAR is a more stable protein than FL-SETMAR, as it more often carries an N-terminal sequence of 13 amino-acids called a-peptide. This suggests a link between exon 2 exclusion (during alternative splicing) and the AUG codon that will be selected for the initiation of the translation. With regard to the mechanism of action of S-SETMAR, a hypothesis formulated by several authors proposes that it could act as a dominant-negative of FL-SETMAR, by competing with partner-proteins, with DNA binding sites, and/or by poisoning FL-SETMAR through heterodimer assembly. However, the occurrence of such heterodimers has never been proven, although their presence could explain some of the ambiguous results described herein for FL-SETMAR. Under this point of view, considering the amount of both proteins within cells seems a reasonable precaution when studying their mechanisms of action.

Against this backdrop of incomplete mechanistic understanding, transcriptomic deregulation mediated by SETMAR proteins varies widely depending on the cellular context and may involve both TIR-associated and non-TIR target genes. Although FL-SETMAR DNA-binding specificity and transcriptional activity through the *Hsmar1* TIR network are now well established [10,13,14,15,16,17], its pleiotropic effects remain highly context-dependent, and the contribution of shorter SETMAR variants has remained largely unexplored. This is particularly relevant in brain-derived cells, since SETMAR is known to play an essential role in primate brain development [4,18].

Here, we aimed to better understand the roles and mechanisms of action of S-SETMAR in glioblastoma cells. We compared differentiated 8MGBA cells with an isogenic line stably over-expressing αS-SETMAR. Our results identify αS-SETMAR as a dual modulator of glioblastoma cell fate, simultaneously slowing proliferation and promoting chromosomal instability while increasing sensitivity to genotoxic stress. These findings support the protective role of αS-SETMAR when over-expressed in glioblastoma patients and suggest that this variant could represent both a prognostic marker and a potential therapeutic tool.

## 2. Materials and Methods

**Biological resources:** 8MGBA (human GB, ACC-432 DSMZ) cells were cultured in Minimum Essential Medium (MEM, Thermo Fisher Scientific, Waltham, MA, USA) supplemented with 10% Fetal Calf Serum (FCS, Thermo Fisher Scientific) and maintained in 5% CO_2_ at 37 °C. For stable transfection, the αS-SETMAR sequence (i.e., including the α-peptide sequence) was cloned downstream of the pEF1 promoter of the pEF1-V5-HisA plasmid (V92020, Invitrogen, Waltham, MA, USA), giving pEF1-V5-αS-SETMAR (plasmid map in Figure S1). Plasmid was transfected in 8MGBA using Fugene^®^ HD (Promega Corporation, Madison, WI, USA), per the manufacturer’s instructions. After 24 h, cells were transferred to 100 mm dishes, and the medium was supplemented with 1200 μg/mL of neomycin (G418, Invitrogen). After 2 weeks of selection, single foci were picked up and grown in a 24-well plate. The stable expression of αS-SETMAR was confirmed by RT-q-PCR and Western blot. The resulting αS-SETMAR-8MGBA cell lines are routinely maintained in MEM supplemented with 10% FCS and 800 µg/µL neomycin. 8MGBA cells containing an empty pEF1-V5-HisA plasmid were similarly prepared and used as a control. In the main text, we use the name “αS-SETMAR” to denote the protein over-expressed in our assays and “S-SETMAR” to denote the short variant described in the literature and for which the presence/absence of the a-peptide is unknown.

**Reagents:** Immunocytochemistry (IHC) for double-strand break (DSB) detection was performed using an anti-γH2AX pS139 antibody (ab11174, 1/1000, Abcam, Cambridge, UK) for the primary antibody, and a donkey anti-rabbit secondary antibody coupled to fluorophore (Alexafluor™ 546, A-10040, 1/500, Thermo Fisher Scientific) for the secondary antibody. IHC for cell proliferation was performed using an anti-Ki67 antibody (ab16667, 1/500, Abcam) for the primary antibody, and a goat anti-mouse secondary antibody coupled to fluorophore (Alexafluor™ 594, A-11005, 1/200, Thermo Fisher Scientific) for the secondary antibody. IHC for spindle analyses was performed using a mouse monoclonal tubulin-a antibody (T6199, 1/250, Sigma-Aldrich, St. Louis, MO, USA) and a secondary donkey anti-mouse antibody (Alexa Fluor 555, red, 1/250), a rabbit polyclonal FL-SETMAR antibody (Ab129455, 1/250, Abcam) and a secondary donkey anti-rabbit antibody (Alexa Fluor 488, green, 1/250) and counterstained with DAPI. For Western blots (WBs), we used the following primary antibodies: anti-SETMAR (Ab129455, 1/2500, Abcam), anti-actin-HRP (A3854, 1/100,000, Sigma-Aldrich), anti-α-peptide (1/1000, custom designed by Covalab, Villeurbanne, France; described in [3]), anti-PARPc (mAb 5625, 1/1000, Cell Signaling Technology, Danvers, MA, USA), and an anti-rabbit IgG, HRP conjugate as a secondary antibody (W401B, 1/2500, Promega Corporation).

**Proteins extraction and Western blot analysis:** Cells were lysed with Radio Immuno-Precipitation Assay (RIPA) buffer (1% NP-40, 0.1% SDS, 0.5% Na-DOC, 20 mM HEPES buffer, pH 7.5, 150 mM NaCl, and 1X HALT inhibitor protease cocktail (Pierce Biotechnology, Rockford, IL, USA)) for 30 min at 4 °C. Cellular debris was removed by centrifugation (13,000× *g* for 10 min at 4 °C), and the supernatant (crude protein extracts) was recovered in a fresh tube. Protein concentrations were assayed using the BCA Protein Assay Kit (DC protein Assay, Bio-Rad Laboratories, Hercules, CA, USA) per the manufacturer’s instructions, using the microplate procedure. For WB assays, 20 mg of protein extract was separated on 4–20% polyacrylamide gels and transferred to nitrocellulose membranes. The immunoblots were revealed using the ECL purity kit (Bio-Rad). Membranes were imaged with Chemidoc Touch equipment (Bio-Rad Laboratories), and signals were quantified with Image J2 software (v2.16.0/1.54p) [19].

**Irradiation:** A total of 100,000 8MGBA or αS-SETMAR-8MGBA cells were seeded per 6-well plate on coverslips for upcoming IHC and irradiated 24 h later in a single dose. An RX-650 irradiator (Faxitron X-Ray Corporation, Wheeling, WV, USA) was used to deliver 1.7 Gy/min. IC experiments were performed 1 h, 7 h and 24 h after irradiation, as indicated. Non-irradiated cells were used as a control.

**Proliferation tests:** A total of 80,000 8MGBA or αS-SETMAR-8MGBA cells were seeded per 4-well chamber slide (Nunc Lab-Tek, Thermo Fisher Scientific, Rochester, NY, USA) for upcoming IHC and incubated in standard conditions for 24 h.

**Immunocytochemistry (IHC):** The cells were fixed with 4% paraformaldehyde (ThermoFisher) and 4% sucrose (Sigma-Aldrich) for 20 min at room temperature (RT), permeabilized in PBS solution containing 0.2% Triton100 (Sigma-Aldrich) for 15 min and blocked in 5% Bovine Serum Albumin (BSA, Sigma-Aldrich) for 20 min. Hybridizations were realized in PBS containing 1% BSA and the primary antibody for 2 h. After washing (in PBS), the cells were incubated in PBS containing 1% BSA and the secondary antibody coupled to fluorophore for 1 h. The cells were then mounted with DAPI ProLong™ Diamond Antifade Mountant solution (ThermoFisher). After drying, immunofluorescence (IF) was imaged with a Nikon Eclipse Ti microscope and captured with a Nikon digital SightDS-Ri1 camera, with a 10× magnification (Nikon Instruments Inc., Tokyo, Japan). Signals from antibodies (anti-Ki67 or anti-γH2AX pS139) were quantified with ImageJ software [19] and normalized against DAPI signals. For spindle analysis, cells were cultured in MEM supplemented with 0.1% FBS for 48 h to induce cell cycle arrest. They were then placed in MEM containing 10% FBS for 24 h before treatment for IHC as described herein. After drying, immunofluorescence (IF) was imaged with a Leica CTR5500 (Leica Microsystems GmbH, Wetzlar, Germany) and captured with a Hamamatsu ImagEM C10600-10B digital camera (Hamamatsu Photonics K.K., Hamamatsu, Japan), with a 63× magnification.

**Growth rates and MTT tests:** A total of 50,000 8MGBA and αS-SETMAR-8MGBA cells were plated in triplicate in 12-wells plates. At 24 h, 48 h, and 72 h, cells were harvested; 10 µL of each well was diluted (1/2) in Trypan Blue, and cells were counted with Kova slides. The number of dead and live cells was assessed, the mean of each replicate was calculated, and the total number of cells was determined. The assay was repeated twice. The remaining cells were used to perform the MTT assay: 40,000 cells were collected and incubated for 1 h at 37 °C in MEM + FCS and MTT (M6494, Invitrogen) at 0.5 mg/mL and centrifugated 5 min at 1100 rpm. The pellet was resuspended in DMSO (the volume was adjusted to obtain DO < 1), transferred to 96-wells plates, and the absorbance at 595 nm was measured.

**Karyotyping and aCGH array studies:** Karyotyping G banding was performed using standard methods on metaphase spreads from the growth phase of 8MGBA and αS-SETMAR-8MGBA cell lines. Genomic DNA was extracted using a Nucleo-Spin Tissue kit (Macherey Nagel GmbH & Co, Düren, Germany). Array comparative genomic hybridization (aCGH) experiments were performed using Agilent Human Genome CGH 180 K oligonucleotide arrays with 8MGBA DNA as a control (Agilent, Santa Clara, CA, USA; https://www.agilent.com (accessed on 12 March 2023)). The custom array had a probe every 13 kb. The arrays were analyzed with the Agilent scanner and Feature Extraction software (v.9.1.3). Graphical overview was obtained using CGH analytics software (v.3.5.14).

**Cell tracking by time-lapse:** A total of 50,000 8MGBA cells and 75,000 αS-SETMAR-8MGBA cells were plated in 6-well plates for 24 h prior to the time-lapse in 3 mL of appropriate culture media. The time-lapse was realized with a microscope equipped with a CO_2_ chamber (Evos M5000, Invitrogen). For 8MGBA cells, pictures were taken every 5 min for 67.25 h, while for S-SETMAR-8MGBA cells, pictures were taken every 10 min for 91.30 h. Throughout the experiment, the chamber was supplied with 5% CO_2_, without hygrometry for 8MGBA cells and 85% for αS-SETMAR-8MGBA cells. The time-lapse was analyzed as previously described [20]. When possible, granddaughters cells were included in the analysis. The cell cycle duration was determined by the time between two anaphases.

**Cell cycle analysis by Fluorescence-Activated Cell Sorting (FACS):** Asynchronous 8MGBA and αS-SETMAR-8MGBA cells were cultured for 24 h and subsequently harvested by trypsinization. The cells were washed with PBS and fixed in 70% ethanol for at least 1 h. After PBS washing, the cells were treated with RNase A (100 μg/mL) and stained with propidium iodide (0.05 mg/mL). Samples were analyzed using a BD FACSMelody™ cell sorter cytometer (Becton, Dickinson and Company (BD), Franklin Lake, NJ, USA). For each condition, 50,000 events were acquired and analyzed. Single cycling cells were gated using FlowJo software (v10.10.1). Cell cycle phases were defined manually because automatic fitting models implemented in the software failed to accurately fit the cell cycle profiles, since these algorithms are optimized for cells displaying canonical cell cycle progression.

**RNA-seq analysis:** RNA was extracted with Direct-Zol RNA miniprep kit (Zymo Research, Irvine, CA, USA), and the quantity was assessed with the Nanodrop 2000 Spectrophotometer (Thermo Fischer Scientific). The quality of RNA was checked with the Agilent 2100 Bioanalyzer, using the RNA 6000 Nano kit (Agilent Technologies, Les Ulis, France), according to the manufacturers’ recommendations. The samples exhibiting an RNA integrity number (RIN) between 8 and 10 (three biological replicates per condition from three independent experiments) were used for the RNA-seq experiment. This work has benefited from the facilities and expertise of Platform Genomics Paris Centre (IBENS). RNA-seq libraries were prepared using a TruSeq Stranded mRNA kit (Illumina, San Diego, CA, USA). Indexed samples were sequenced on a Nextseq Illumina 500 to obtain single-end reads. Low-quality sequences were improved with Trimmomatic (Galaxy Version 0.38.0). Reads were mapped on the human reference genome GRCH38.p11 with HISAT2 (Galaxy Version 2.2.1 + galaxy0). The expression level of transcripts was quantified with htseq-count (Galaxy Version 0.9.1), and differential expression was performed with DESEQ2 (Galaxy Version 2.11.40.8). Differentially expressed genes (DEGs) with an adjusted *p*-value < 0.05 and |Log2FC| > 1 were retained for functional analysis. For data analysis, gene lists were recovered using the NCBI (https://www.ncbi.nlm.nih.gov/gene (accessed on 1 July 2023)) and the GO (https://geneontology.org/ (accessed on 1 July 2023)) datasets to upload *Homo sapiens* genes involved in various cellular processes (cell cycle; G1, S, G2, and M phases; G1/S, S/G2, G2/M, metaphasis checkpoints, spindle assembly, and chromosome segregation). Gene lists are shown in Table S1.

**Apoptosis assays:** A total of 10^6^ 8MGBA and αS-SETMAR-8MGBA cells were cultured in T25 flasks for 24 h and then treated (or not, for the control) with 1 mM of doxorubicin (Sigma, D1515-10MG) in 5 mL of fresh medium for 48 h. The treated cells and respective controls were collected, and the cell pellets lysed in RIPA lysis buffer to extract proteins and measure the appearance of cleaved-PARP (PARPc) as apoptosis landmarks, by WB. Each assay was repeated three times.

**Statistical analyses:** In the RNA-seq procedure, *p*-values were adjusted for multiple testing using the Benjamini–Hochberg method, which controls the false discovery rate (FDR), to obtain statistical estimates. Other statistical analyses were performed using GraphPad Prism 11 as described in the main text.

## 3. Results and Discussion

Here, we used the name “αS-SETMAR” to denote the protein over-expressed in our assays and “S-SETMAR” to denote the short variant describe in the literature and for which the presence/absence of the a-peptide is unknown. Since it greatly increases αS-SETMAR half-life when compared to S-SETMAR, its presence is therefore a factor influencing the relative quantity of S-SETMAR within the assays. To study the role of αS-SETMAR, we have stably modified the 8MGBA cell line to create a line called αS-SETMAR-8MGBA, which constitutively expressed αS-SETMAR in amounts greater than FL-SETMAR (Figure S2). Several clones were recovered, and the one with the strongest αS-SETMAR expression (clone 1 in Figure S2) was used for further work.

### 3.1. αS-SETMAR and DNA Repair

As mentioned above, the efficiency of S-SETMAR in NHEJ has been already assayed in vitro, using a fusion protein (MBP-S-SETMAR) [9]. As a result, S-SETMAR was found to poorly sustain NHEJ compared to FL-SETMAR. To verify whether these findings correctly reflect what happens in a cellular context, X-rays irradiation (IR) was first tested using three single doses (2, 5 and 10 Gy) to determine which one should be used. For both cell lines, a decrease in cell viability (87% for 8MGBA and 83% for αS-SETMAR-8MGBA cells) was only seen for the 10 Gy dose at 24 h. In addition, this dose induced DNA damage visible by IF γH2AX labeling but did not induce cell death even after 6 days post-IR.

**Figure 1:**
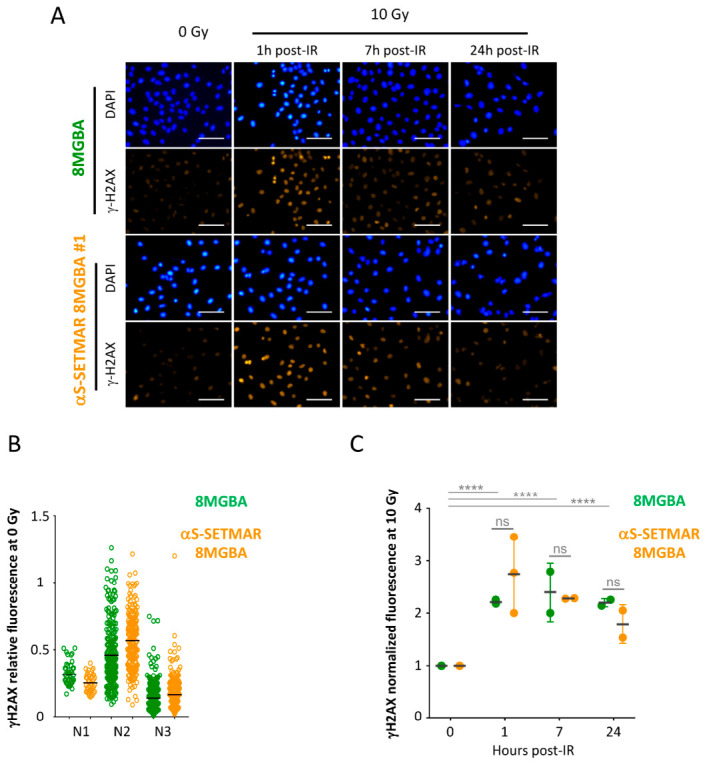
αS-SETMAR does not modify DSB repair. (**A**) γ-H2AX foci revealed by IF in 8MGBA and αS-SETMAR-8MGBA cells without irradiation (0 Gy) and 1, 7 and 24 h post-irradiation (10 Gy). Scale: 100 μm. (**B**) Quantification of γ-H2AX foci fluorescence intensity at 0 Gy in 8MGBA (green dots) and αS-SETMAR-8MGBA (orange dots) cells, using ImageJ software. Scatter plots of all points are shown for each assay (N). Means are shown as black bars. Within each assay (N), statistical analyses reveal no differences between both cell lines (Wilcoxon matched-pairs signed-rank test, two-tailed). (**C**) Quantification of γ-H2AX foci fluorescence intensity at 1, 7 and 24 h post-irradiation (10 Gy) in 8MGBA (green dots) and αS-SETMAR-8MGBA (orange dots) cells, using ImageJ software. For each point post-irradiation (time and cell line), the mean fluorescence intensity is calculated within each assay (N1, N2 and N3) and normalized against the mean value at 0 Gy of the same assay. For each time post-irradiation, statistical analyses reveal no differences between the two cell lines (Wilcoxon matched-pairs signed-rank test, two-tailed). **** indicates a *p*-value < 0.0001 (Wilcoxon matched-pairs signed-rank test, two-tailed), for both cell lines between 0 and the corresponding post-irradiation time.

IR was thus performed at 10 Gy on 8MGBA cell lines over-expressing or not over-expressing αS-SETMAR. γ-H2AX foci were detected by IF at 0, 1, 7 and 24 h post-IR, and the extent of DNA repair was evaluated by the overall decrease in foci intensity [21] (Figure 1A). We first verified that the overexpression of αS-SETMAR does not modify the cellular background of double-strand breaks (DSBs). Three independent assays were performed (N1, N2, and N3). For each assay and each cell line, a large variability in the relative fluorescence intensity (γ-H2AX/DAPI) of foci was observed, reflecting the variability of each test. Indeed, the mean of each assay revealed no significant difference between the two cell lines (Figure 1B). We assumed that αS-SETMAR does not modify the cellular background of DSBs. We then analyzed the kinetics of DSB repair for both cell lines. To circumvent the bias due to experimental internal variability previously mentioned, the mean of γ-H2AX relative fluorescence detected at 0 Gy for each assay was normalized to 1 (as the reference). The mean of γ-H2AX relative fluorescence detected at each time after IR was expressed relative to its own reference. This allowed us to obtain the γ-H2AX normalized fluorescence at 10 Gy (Figure 1C). We showed that the number of DSBs increased by a factor of two to three after 1 to 7 h (*p* < 0.0001) for both cell lines, with no significant difference between αS-SETMAR 8MGBA and 8MGBA. At 24 h post-IR, the level of DSBs was still twice that of 0h (*p* < 0.0001) for both cell lines, again with no significant difference between them. In conclusion, αS-SETMAR does not significantly modify the overall DSB repair. Although the method used is less precise than a quantitative per-nucleus analysis, it nevertheless provides evidence supporting the lack of efficiency of αS-SETMAR in NHEJ, as previously demonstrated [9].

### 3.2. αS-SETMAR Reshapes Cell Cycle Dynamics

Despite numerous studies, the role of FL-SETMAR in cell proliferation remains unclear, with conflicting results likely reflecting major differences in experimental design. Endogenous depletion strategies targeting the MAR domain—thereby affecting all SETMAR variants—consistently led to reduced proliferation [5,6,7]. In contrast, FL-SETMAR overexpression was reported to increase proliferation in HEK-293T cells, which lack endogenous SETMAR expression [6], whereas no proliferative effect was observed in U2OS cells; instead, proliferation decreased in proportion to FL-SETMAR levels [8]. Notably, endogenous SETMAR expression was not assessed in this latter context. Finally, endogenous FL-SETMAR appears absent from healthy and tumoral colorectal tissues, where shorter MAR-only variants predominate [10].

Considering the SETMAR network and the possible presence of both variants within cells, we assumed that (1) the relative level of both variants may have an impact on cell growth, and (2) each variant may have a different effect. We thus decided to analyze the growth of 8MGBA cells under two conditions: control cells, in which FL-SETMAR is predominant (FL > S), and recombinant αS-SETMAR-8MGBA cells, in which αS-SETMAR is very strongly predominant (S >> FL). As a preliminary step, we checked whether aS/FL-SETMAR heterodimers could assemble in cells co-expressing both variants, a hypothesis that has never been formally demonstrated. Our results (Figure S3) indicate that such heterodimers may exist, but they appear rare and/or relatively unstable, even less stable than the FL/FL or αS/αS homodimers. The impact of these heterodimers has therefore been neglected in the rest of our study. Next, we checked for αS-SETMAR overexpression effects in cell proliferation.

**Figure 2:**
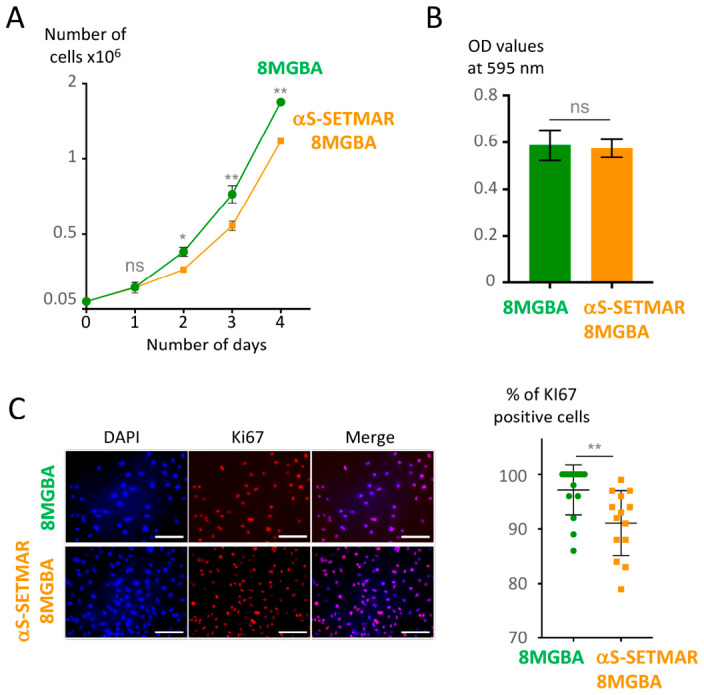
αS-SETMAR modulates cell behavior. (**A**) Growth curves of 8MGBA (green) and αS-SETMAR-8MGBA (orange) cells grown in standard conditions. From the second day, the number of αS-SETMAR-8MGBA cells was significantly lower than that of 8MGBA (N = 6; Mann–Whitney, two-tailed, at J2, *p* * = 0.015; J3, *p* ** = 0.0087; J4, *p* ** = 0.0022). (**B**) MTT assay of 8MGBA (green) and αS-SETMAR-8MGBA (orange) cells. Histograms show the means of OD values at 595 nm, with no differences between cell lines (N = 3, Mann–Whitney test, two-tailed). (**C**) Proliferation of 8MGBA (green) and αS-SETMAR-8MGBA (orange) revealed by Ki67 IHC staining. Scatter dot plots show the percentage of Ki67-positive cells in both cell lines (n = 15, *p* ** = 0.0014 in Mann–Whitney test, two-tailed). Scale: 100 μm.

After two days of culture, we observed a small but significant drop in the number of cells for the αS-SETMAR-8MGBA cell line. This difference increased with time (Figure 2A), suggesting that αS-SETMAR has slowed down cell proliferation, either directly or indirectly. To investigate the underlying mechanisms, we verified at first if αS-SETMAR decreased 8MGBA viability and/or proliferation. MTT tests revealed no detectable differences between the two cell lines’ viability (Figure 2B), whereas Ki67 labelling revealed a small (about 8%) but significant (*p* = 0.0014) decrease in αS-SETMAR-8MGBA cell proliferation when compared to 8MGBA cells (Figure 2C), in agreement with the slight but significant effect seen in Figure 2A.

**Figure 3:**
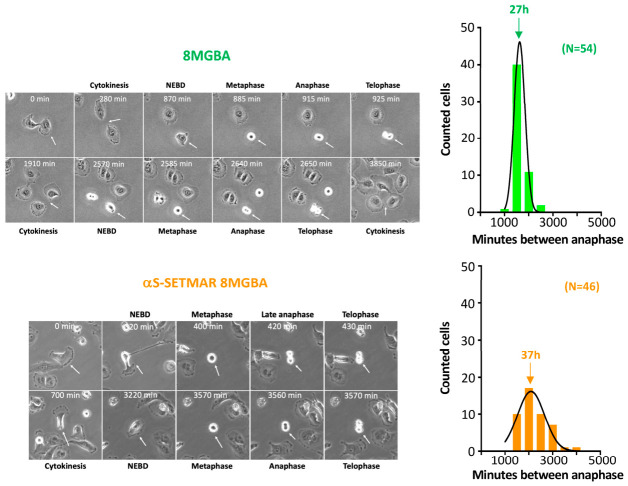
αS-SETMAR modifies the cell cycle duration. Time-lapse of 8MGBA (green, N = 54) and αS-SETMAR-8MGBA (orange, N = 46) cells grown in standard conditions. The time between two anaphases was counted in minutes for each cell line. Representative images of one cell cycle are shown (left panels, 20x magnification), with an arrow indicating the cell being monitored. In the right panels, each histogram bar shows cells that take from 1000 to 3000 min to complete a full cycle (8MGBA, green bars), with one category every 500 min (1000–1500; 1500–2000; 2000–2500; 2500–3000) and from 1500 to 4500 min to complete a full cycle (αS-SETMAR-8MGBA, orange bars), with one category every 500 min (1500–2000; 2000–2500; 2500–3000; 3000–3500; 3500–4000; 4000–4500). Extrapolation curves are shown as black lines, with the top of the curves indicating the average cycle time for each line, converted to hours. Distributions and related curves are statistically different (Mann–Whitney *p* < 0.0001).

These observations prompted us to further investigate whether αS-SETMAR affects cell cycle progression. We first carried out time-lapse assays covering a period assumed to be necessary to complete at least two to three full cycles, i.e., 96 h (Figure 3, left panels, and Figure S4, time-lapse motions).

We observed that 8MGBA cells need 27 h to complete a full cycle (measured between the anaphase of a mother cell and that of its daughter cells), while αS-SETMAR-8MGBA needed 37 h to complete the same process (Figure 3, right panel, and Figure S4, time-lapse motion). This observation is sufficient to explain why αS-SETMAR-8MGBA cells’ growth was lower (Figure 2) but raises the question of whether a particular phase of the cycle was affected or not.

**Figure 4:**
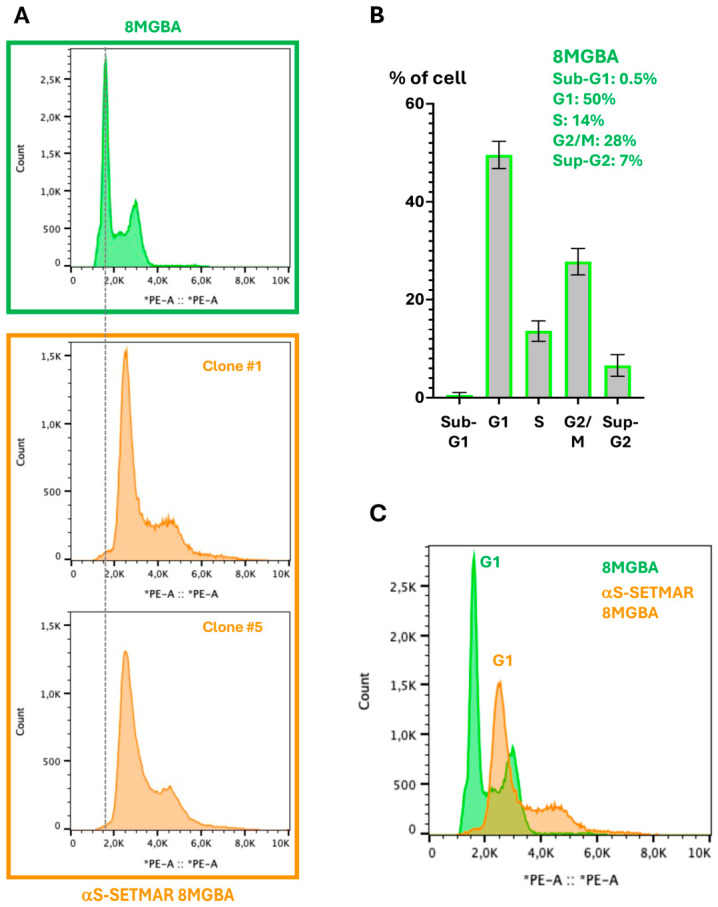
αS-SETMAR modifies the cell cycle profile. (**A**) FACS analysis of 8MGBA (green: N = 3, n = 9) and αS-SETMAR-8MGBA (orange: N = 8, n = 14) cells, using propidium iodide (PI) staining. Typical histograms are shown, including two independent αS-SETMAR-8MGBA cell lines. Similar distributions were obtained with Vybrant DyeCycle Violet, confirming the robustness of the analysis. The vertical dotted line indicates the position of the G1 peak in 8MGBA control cells. (**B**) Cell cycle distribution of 8MGBA was determined by manual gating after exclusion of debris. Owing to aneuploid DNA profiles, model-based fitting algorithms (e.g., Dean–Jett–Fox and FlowJo) were not suitable. Percentages correspond to gated populations. (**C**) Stacked FACS analysis of αS-SETMAR-8MGBA and 8MGBA control cells, with the same color code: 8MGBA (green) and αS-SETMAR-8MGBA (orange).

**Figure 5:**
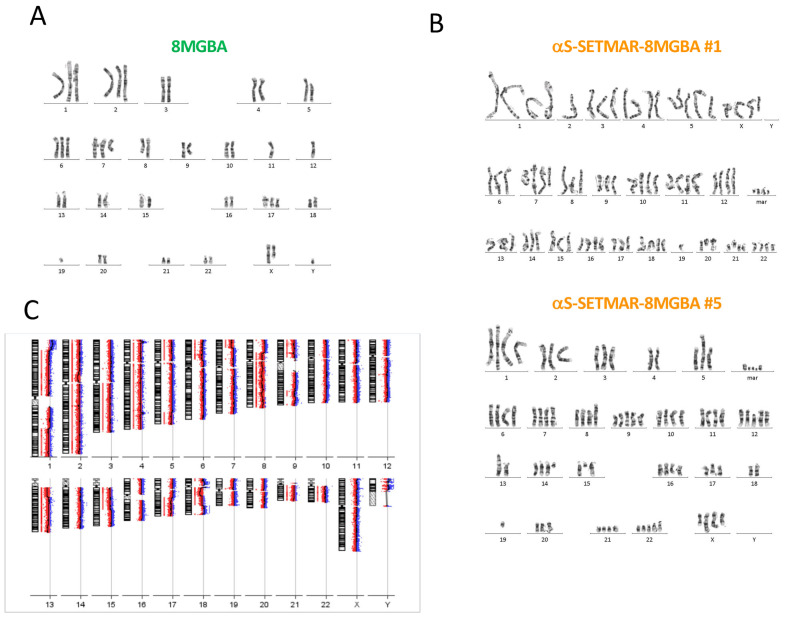
αS-SETMAR triggers aneuploidy. (**A**) Karyotyping of 8MGBA cells. (**B**) Karyotyping of αS-SETMAR-8MGBA clone 1 and clone 5. (**C**) CGH array analysis of αS-SETMAR-8MGBA clone 1 using 8MGBA as a control. Red: deletions; Blue: duplications.

To address this question, we performed FACS analyses (Figure 4A). The histogram distribution observed for the 8MGBA cell line was consistent with that expected for cycling human cancer cells, allowing cell cycle phase quantification (Figure 4B). This analysis was performed by manual gating after exclusion of debris, as DNA content profiles were not compatible with reliable model-based fitting, consistent with the aneuploid status of these cells (see Figure 5A). It indicated that 50% of cells were in G1, 14% in S, and 28% in G2/M. These values fall within the range commonly reported for asynchronously proliferating mammalian cells [22], although the proportion of cells in G2/M was relatively elevated, consistent with the altered G2/M progression frequently observed in cancer cell lines.

In contrast, αS-SETMAR-expressing cells display markedly altered DNA content profiles, characterized by poor resolution between S and G2/M phases and a prominent shoulder on the left side of the G1 peak (sub-G1). The absence of a clearly defined G2/M peak, together with the overall distortion of the DNA content distribution, precludes reliable global quantification of cell cycle phase distribution using standard fitting approaches. Attempts at manual gating by independent operators confirmed the robustness of two features: an increased sub-G1 population (2% versus 0.2%, *p* < 0.0001 in Mann–Whitney two-tailed test) and a modest but reproducible increase in the G1 fraction (53% versus 49%, *p* = 0.0028 in Mann–Whitney two-tailed test) in αS-SETMAR-expressing cells compared to 8MGBA cells, whereas S-phase estimates were variable between experimenters, and G2/M appeared reduced overall (Figure S5). In contrast, no reproducible difference was observed for the supra-G2 population between conditions. Altogether, these observations indicate that αS-SETMAR expression is associated with a global alteration of cell cycle progression, characterized by a relative accumulation of cells in G1 and a loss of normal post-G1 DNA content structure, suggesting a global dysregulation of cell cycle organization rather than a simple redistribution between canonical S and G2/M phases.

FACS analyses revealed another feature that cannot be solely explained by the prolonged cell cycle of αS-SETMAR-8MGBA cells. Specifically, the G1 peak showed a consistent shift toward higher fluorescence intensity in αS-SETMAR-8MGBA cells compared to 8MGBA cells, consistent with an overall increase in DNA content estimated to be on the order of 1.5- to 2-fold (Figure 4C). This shift was most apparent for the G1 population, as S and G2/M phases were poorly resolved in αS-SETMAR-8MGBA cells.

Together, these observations indicate that αS-SETMAR expression induces a global disruption of cell cycle progression rather than a discrete phase-specific arrest. The marked alterations in DNA content profiles further suggest defects in genome maintenance and DNA content homeostasis. However, cell cycle analyses alone do not allow direct assessment of the impact of αS-SETMAR on genome integrity. We therefore investigated this question using karyotype analysis and comparative genomic hybridization (aCGH).

### 3.3. αS-SETMAR Triggers Genome Chaos

8MGBA cells are known to be hyper-diploid cells with about 15% aneuploidy, containing 47–52 chromosomes (DSMZ, https://www.dsmz.de/ (accessed on 1 June 2023)). 8MGBA control cells conformed to this description since they contained 49 chromosomes (Figure 5A). In contrast, αS-SETMAR-8MGBA cells contained 77 to 80 chromosomes (Figure 5B), thus displaying an increased aneuploidy correlated to their greater amount of DNA (×1.6). It is worth noting that karyotypes of αS-SETMAR-8MGBA cells were difficult to establish, as chromosomes were largely broken and modified. However, comparison of both αS-SETMAR-8MGBA clones (1 vs. 5) revealed that the aneuploidy did not concern the same chromosomes, suggesting either that an original event with variable consequences had occurred for each newly established clone (during the production of stable cell lines) or a still non-stabilized genome within each cell line.

aCGH was then performed (using controlled 8MGBA cells as the reference) to analyze which regions were amplified or deleted (Figure 5C). Thus, in addition to aneuploidy, aCGH analysis revealed that αS-SETMAR-8MGBA cells had undergone a profound reorganization of chromosomal structure. The data presented in Figure 5 suggest two major types of rearrangements: translocations and variations in gene copy number.

Over the past fifteen years, catastrophic genomic events resulting in multiple complex chromosomal rearrangements, collectively referred to as *chromoanagenesis*, have been proposed to occur at one or more loci during the cell cycle. Some of these events arise from replication errors, whereas others occur during mitosis, two stages that seem to be affected by αS-SETMAR overexpression (Figure 4). Translocations involving more than two chromosomes, known as *chromoplexy* [23], do not alter the gene copy number. In contrast, *chromoanasynthesis* and *chromothripsis* are typically associated with copy number variations. In chromoanasynthesis, replication defects promote amplifications (duplications/triplications) and deletions of large chromosomal regions. Chromothripsis, by contrast, occurs during mitosis when a chromosome arm is shattered into numerous DNA fragments that are then imperfectly reassembled. During this restoration process, fragment orientation can change; some fragments are lost, while others may form extrachromosomal double minutes. In this scenario, no net increase in copy number is expected. However, this field remains under active investigation, and some studies have suggested that chromothripsis may occasionally result in a moderate copy number gain (two to three copies) [24]. These large-scale genomic rearrangements are thought to arise from a single catastrophic event [25], challenging the traditional view of tumorigenesis as a gradual accumulation of mutations. Instead, they may enable the rapid acquisition of hundreds of rearrangements within only a few cell divisions. Furthermore, chromothripsis appears to occur more frequently than initially anticipated, being detected in more than 50% of several cancer types, including glioblastoma [24]. Our data revealed that all αS-SETMAR-8MGBA chromosomes were affected, indicating widespread chromosomal instability and extensive structural rearrangements, hereafter referred to as “chromosomal chaos”.

### 3.4. αS-SETMAR and Cell Death

**Figure 6:**
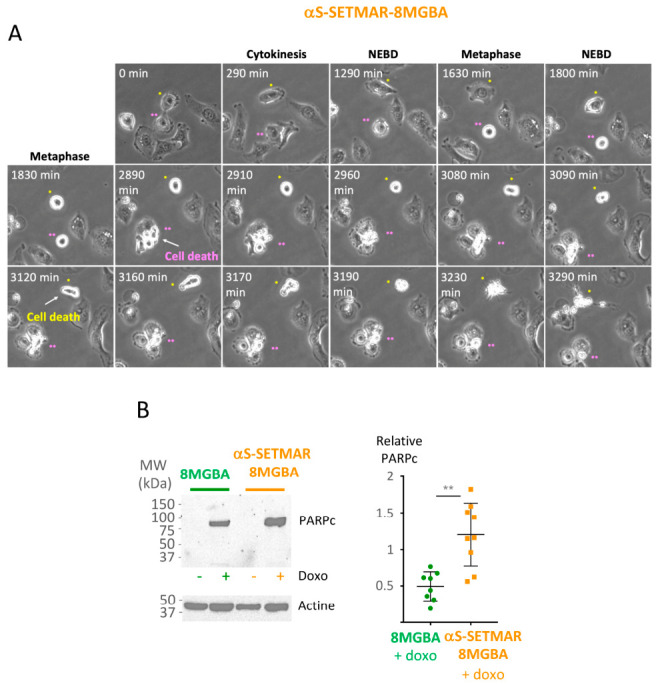
αS-SETMAR and cell death. (**A**) Time-lapse of αS-SETMAR-8MGBA cells grown in standard conditions, with a focus on two cells which remained stuck in mitosis for 21 h before dying. The corresponding cells are marked with white stars before mitosis and pink or yellow stars after (×20 magnification). (**B**) Apoptosis assay of 8MGBA (green) and αS-SETMAR-8MGBA (orange) cells. PARPc signals were detected by Western blot for cells treated (+) or not (-) with 1mM doxorubicin (Doxo). Scatter dot plots show the normalized PARPc signal for both cell lines treated with doxorubicin (N = 8, *p* ** = 0.0025 in Mann–Whitney test). The whole blots (uncropped blots) are shown in the Supplementary File.

An intriguing question for the scientific community studying chromosomal chaos concerns how cells can survive such crises, which would normally lead to cell death through apoptosis or autophagy. This type of cell fate was indeed observed during time-lapse imaging: in αS-SETMAR-8MGBA cells, but not in 8MGBA cells, some cells failed to progress through mitosis. They appeared arrested, sometimes for several hours (approximately 21 h for the two cells marked with stars in Figure 6A and Figure S4, time-lapse motion), before ultimately disintegrating. Consistently, FACS analyses of αS-SETMAR-expressing cells revealed an increased sub-G1 population, indicative of DNA fragmentation and consistent with ongoing cell death under these conditions.

We therefore examined the extent to which both cell lines undergo apoptosis, under standard growth conditions or following induction. In the absence of apoptosis induction, the level of cleaved PARP (PARPc, an apoptosis marker) (Figure 6B, left panel) revealed no differences between control 8MGBA cells and those over-expressing αS-SETMAR, with no signal for both cells. This is not unexpected, as apoptotic events detected by FACS and time-lapse imaging in αS-SETMAR-expressing cells are rare under basal conditions and therefore not readily detectable by immunoblot analysis. However, the results were different if cells were treated beforehand with 1 mM doxorubicin for 48 h to induce a high level of DNA double-strand breaks (i.e., these conditions differed from those used during irradiation, where cells suffered a DNA double-strand break level that triggers repair mechanisms not apoptosis). Here, the PARPc signal was significantly higher (*p* = 0.0025) in αS-SETMAR-8MGBA cells when compared to 8MGBA cells (Figure 6B, right panel). Collectively, these results indicate that αS-SETMAR expression is associated with a low but detectable level of basal apoptosis, as revealed by single-cell approaches (FACS and time-lapse imaging). Importantly, αS-SETMAR-expressing cells display a significantly enhanced apoptotic response upon genotoxic stress, highlighting an increased susceptibility to DNA-damage-induced cell death in this context.

We hypothesized that the phenotypes described so far could reflect broader alterations in cellular programs controlling replication stress responses, cell cycle regulation, and genome maintenance. We therefore performed transcriptomic analyses to identify molecular pathways associated with αS-SETMAR overexpression and to gain insight into the regulatory programs underlying the observed cellular phenotypes.

### 3.5. Transcriptomic Landscape of αS-SETMAR-Expressing Cells

**Figure 7:**
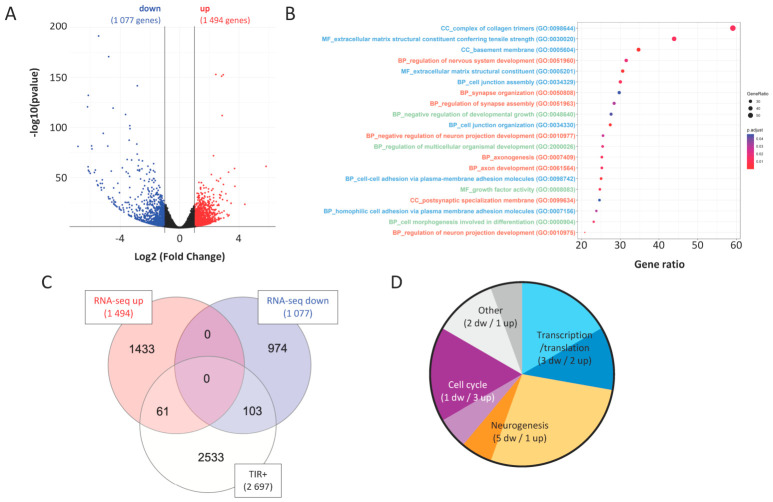
RNA-seq data. (**A**) Volcano plots of DEGs (αS-SETMAR-8MGBA vs. 8MGBA). (**B**) Gene ratio of enriched GO pathways. (**C**) Venn diagram of αS-SETMAR up-regulated genes (red), αS-SETMAR down-regulated genes (blue) and *Hsmar1* TIR-containing genes (white). (**D**) αS-SETMAR deregulated genes (18) that were also found to be targeted by FL-SETMAR in ChIP-seq analysis are classified by main function: neurogenesis (dark orange, up DEGs; clear orange, down DEGs), cell cycle (dark purple, up DEGs; clear purple, down DEGs), and transcription/translation only (dark blue, up DEGs; clear blue, down DEGs).

Differential expression data indicated that αS-SETMAR overexpression modified the expression of about 2500 genes (with |LogFC| > 1, *p* < 0.05), for a total of 1494 up-regulated and 1077 down-regulated genes (Figure 7A). All gene lists used in this study have been compiled in Table S1. Gene ontology analysis revealed an enrichment in three main groups related to extracellular matrix/cell junctions (307 genes), synapses/axons organization (210 genes) and development/growth regulation (184 genes) (Figure 7B). These data were consistent with previously published results. Of note, 2697 genes in the human genome contain *Hsmar1* TIRs, providing context for the subset of TIR-containing differentially expressed genes (DEGs) identified in αS-SETMAR-8MGBA cells (Figure 7C).

We then took advantage of the two previous studies that were done in human colorectal cancer cells. In each study, a different SETMAR variant was analyzed by ChIP-seq: recombinant FL-SETMAR in U2OS cells [14] and endogenous S-SETMAR in HT29 cells, which is, to our knowledge, the only reported cell line expressing only this SETMAR isoform and endogenous S-SETMAR in HT29 cells, which is, to our knowledge, the only reported cell line expressing only this SETMAR isoform [10]. The available data indicated that 615 genes were targeted by FL-SETMAR (in U2OS cells) and 3238 by S-SETMAR (in HT29 cells). These values did not necessarily mean that there were more target genes for S-SETMAR than for FL-SETMAR (although this cannot be ruled out), but rather reflected the difference between both experiments, in particular the use of different antibodies and the low level of recombinant FL-SETMAR expression (as specified by the authors). We identified (with a Venn diagram) 359 genes that were bound by both proteins, while 256 were only bound by FL-SETMAR and 2878 only by S-SETMAR (Table S2A). These results suggest that some genes were possibly bound by any isoform of the SETMAR proteins, while others seem to be preferentially bound by one or the other. The most amazing point revealed by gene ontology was that target genes of either S-SETMAR or both proteins were mainly involved in central nervous system (CNS) development (Table S2B), even though the cells used for the experiment are not CNS cells. In contrast, no specific enrichment was found for the genes targeted by FL-SETMAR, perhaps due to the low number of genes. Altogether, these observations confirm the involvement of SETMAR in the human embryo brain development, as already proposed [4], while suggesting that this role is mainly played by S-SETMAR.

#### 3.5.1. Dysregulation of Cell Cycle Regulatory Networks

**Figure 8:**
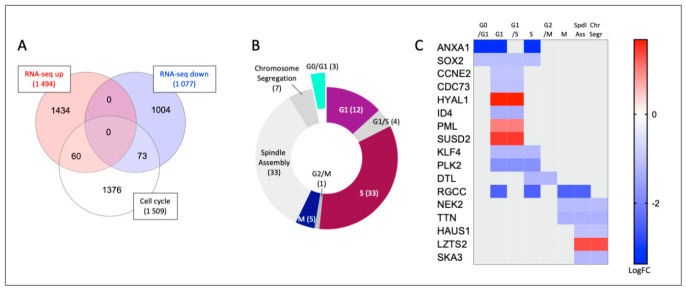
αS-SETMAR deregulated genes involved in the cell cycle. (**A**) Venn diagram of αS-SETMAR up-regulated genes (red), αS-SETMAR down-regulated genes (blue) and cell cycle genes as retained from Table S2 (white). (**B**) Numbers of αS-SETMAR deregulated genes involved in phases and checkpoints of the cell cycle. S/G2 and G2 are omitted because no deregulated genes were found associated with them. (**C**) Heat-map of the 17 αS-SETMAR deregulated genes (left margin) involved in more than one stage of the cell cycle (top of the figure). The color code is shown on the right, for up-regulated genes (red) and down-regulated genes (blue).

To further characterize the mechanisms underlying altered cell cycle progression in αS-SETMAR-expressing cells, we analyzed DEGs involved in cell cycle regulation and checkpoint control. Genes involved in cell cycle were selected by combining NCBI (https://www.ncbi.nlm.nih.gov/gene (accessed on 1 July 2023)) and GO (http://geneontology.org (accessed on 1 July 2023)) datasets. Only the common ones (1509) were retained for further analysis (Table S1). Among these, 60 were up-regulated by αS-SETMAR and 73 down-regulated when compared with our RNA-seq data (Figure 8A).

We then investigated these genes’ distribution in the different phases of the cell cycle and the main checkpoints. As performed for the whole cell cycle, gene lists from each phase and/or checkpoint were retrieved (Table S1) and compared with the DEGs. Consistent with the altered cell cycle structure observed by FACS analyses, transcriptomic profiling revealed deregulation of genes involved in S phase progression, DNA replication, and mitotic spindle assembly. Analysis of differentially expressed genes (DEGs) (Figure 8B) showed that multiple cell-cycle processes were affected, with notable contributions from G1 (12 DEGs), S phase/replication (11 and 22 DEGs, respectively), a strong enrichment in spindle assembly (33 DEGs), and a more limited involvement of chromosome segregation pathways (seven DEGs). Overall, these processes accounted for 50 DEGs, while cell-cycle checkpoint-related genes accounted for 48 DEGs. Several genes were assigned to more than one category; after removing redundancies, 66 distinct genes were identified, distributed across the entire cell cycle. Given that FACS analyses did not allow the identification of a specific phase preferentially affected by αS-SETMAR overexpression (Figure 4), we next considered cell-cycle-wide effects and examined genes involved across all stages of the cell cycle.

Overall, 17 genes drew attention (Table S3), because they were described as key regulators of the whole cell cycle and identified here in two steps or more (Figure 8C). The four most deregulated genes were related to the interphase.

*ANXA1* (known as an effector of glucocorticoid-mediated responses and a regulator of the inflammatory process) was involved in multiple cancer processes, including cell proliferation. In GB, *ANXA1* was shown to be overexpressed and negatively associated with poor survival. A recent study [26] reported that its down-regulation suppressed GB cell proliferation, in line with our findings. Among the 17 genes retained here, *ANXA1* was the most down-regulated (LogFC = −3.3), accounting for the antiproliferative effect related to αS-SETMAR.

Hyaluronan, the major component of the extracellular matrix, was thought to be involved in cell proliferation, migration and differentiation and thus may play a role in promoting tumor progression. GB cells have been shown to overproduce hyaluronan, which in turn may form a halo around the cells, inducing dendritic cell death and generating an immune-protective barrier for the tumor [27]. In our study *HYAL1* (coding for a lysosomal hyaluronidase that intracellularly degraded hyaluronan) is overexpressed in αS-SETMAR-8MGBA cells (LogFC = 1.67). It is tempting to propose that HYAL1 overexpression could prevent hyaluronan overproduction and therefore limit its related proliferative effects.

In cancer, *PLK2* (a gene from the polo family of serine/threonine protein kinases) is generally considered to be a tumor suppressor, whereas its role in GB is more ambiguous. Recent findings [28] underline that the low expression of *PLK2* in GB is due to DNA hypermethylation and predicts favorable prognosis. From the point of view of its mechanism of action, PLK2 is essential for mitotic centriole replication, and its loss leads to cell cycle disorders. In our data, *PLK2* was down-regulated by αS-SETMAR overexpression (LogFC = −1.8), in line with the protective effect of S-SETMAR.

The fourth gene, *SUSD2,* involved in neuritic outgrowth and excitatory synapse numbers [29], was found to be significantly down-regulated in cancers, and to function as a tumor suppressor. Upon ligand binding, this membrane protein induced G1 cell cycle arrest through inhibition of the cyclin D/CDK6 complex [30]. Overexpression of *SUSD2* in αS-SETMAR-8MGBA cells (LogFC = 1.46) was also in line with the protective effect of S-SETMAR.

To finish, the fifth gene was linked to the whole cycle. *RGCC* is known to regulate cell cycle progression in many cell types. In the human brain, its expression levels were related to the fate of neural stem cells (NSCs) during cortical development between neural [31] and glial lineages [32]. In addition, *RGCC* KO disrupted the centrosome and spindle organization of NSCs during mitosis and impaired the expression of essential centrosomal proteins. In gliomas, RGCC has also been found located at the centrosome [33], as is believed for FL-SETMAR, albeit in another cell type [14]. *RGCC* was shown to be down-regulated in GB [34] but overexpressed in many cancers. Finally, the hypothesis that *RGCC* could function either as a tumor suppressor or promoter depending on cellular context [35] made it difficult to predict its role in 8MGBA cells. However, the strong down-regulation of *RGCC* in αS-SETMAR-8MGBA cells (LogFC = −2.48) may impair mitosis through spindle and/or centrosome mis-organization.

Together, the deregulation of these five genes may contribute to the prolonged cell cycle duration and broader cell cycle alterations observed in αS-SETMAR-8MGBA cells, in agreement with the proposed protective role of S-SETMAR in GB.

#### 3.5.2. Transcriptional Signatures of Mitotic and Chromosomal Instability

As previously noted, aCGH analysis revealed that αS-SETMAR-8MGBA cells had undergone a profound reorganization of chromosomal structure, leading to variations in gene copy number. We emphasize that the differentially expressed genes examined here were verified not to reside within regions of copy number amplification or deletion, indicating that the observed changes reflect genuine transcriptional deregulation rather than gene dosage effects. To illustrate this point, a detailed aCGH profile of chromosomes carrying the differentially expressed genes of interest identified earlier (*ANXA1*, *HYAL1*, *PLK2*, *SUSD2*, and *RGCC*) is provided in Figure S6.

In agreement with the chromosomal alterations detected by karyotype and aCGH analyses, transcriptomic profiling revealed a marked enrichment of genes associated with chromosome segregation and mitotic spindle organization. Notably, a large proportion of DEGs identified in Figure 8B were linked to S phase and mitosis, with a particular enrichment in spindle assembly pathways, suggesting a potential connection between these transcriptional changes and the chromosomal instability observed in αS-SETMAR-expressing cells. We therefore hypothesized that αS-SETMAR expression may impair proper spindle formation during mitosis.

After removing redundancies, we obtained a set of 35 unique genes (Table S4A), representing 53% of the 66 cell-cycle-related DEGs. These genes are likely to influence this critical stage of mitosis, primarily through pathways governing spindle assembly and centrosome dynamics. These 35 genes were manually curated to verify their genuine involvement in the cell cycle, excluding those not actually implicated. The resulting dataset (Table S4A, pink lines) comprised 31 genes whose deregulation upon αS-SETMAR overexpression was associated with marked chromosomal instability. For 8 of the 31 genes, RNA-seq LogFC variations could not be unambiguously attributed to αS-SETMAR overexpression, as they correlated with copy number changes detected by aCGH in αS-SETMAR-8MGBA cells relative to the control 8MGBA line. The remaining 23 genes were therefore considered unaffected by copy number variation, and their RNA-seq changes were attributed solely to αS-SETMAR overexpression. Notably, 19 of these 23 genes are directly involved in spindle assembly and/or centrosome-related functions (Table S4B, blue lines). This set included three genes already discussed as players in centriole, centrosome or spindle functions: *ANXA1* allows mitotic spindle orientation during mammalian epithelial morphogenesis [36], *PLK2* is essential for mitotic centriole replication [37], and the protein RGCC has been found at the centrosome [33].

To directly assess the involvement of αS-SETMAR in spindle assembly, we performed IF analyses on both 8MGBA and αS-SETMAR-8MGBA cells to visualize spindle architecture.

**Figure 9:**
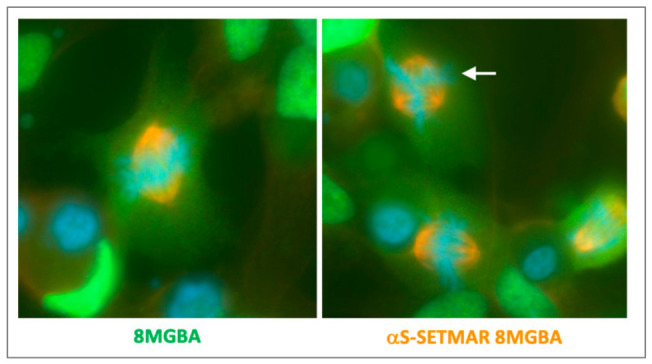
αS-SETMAR promotes tripolar spindles. Spindles revealed by IF in 8MGBA and αS-SETMAR-8MGBA cells. DNA: blue; Tubulin-α: orange; SETMAR: green. No abnormal spindle was ever observed in the 8MGBA lineage, whereas some cells in the αS-SETMAR-8MGBA lineage clearly show a tripartite spindle, as indicated by the white arrow.

Notably, tripolar spindles were observed in approximately 30% of αS-SETMAR-8MGBA cells, whereas they were completely absent in 8MGBA cells (Figure 9), further supporting a role for αS-SETMAR in promoting chromosomal chaos. Although this fraction appears substantial, these events are likely distributed over time rather than occurring synchronously across the cell population. Consequently, the continuous elimination of cells undergoing mitotic catastrophe may coexist with an apparently preserved overall viability. In parallel, a lengthening of the cell cycle is observed. At the present stage, these two phenomena cannot be formally linked, but they may independently contribute to the overall decrease in proliferation.

Alternatively, altered chromosome segregation can result from defects in centromere organization. In a recent review, Samejima et al. [38] listed 367 key centromeric proteins, among which 11 of our 19 DEGs were included (marked with a star in Table S4B). It should be noted, however, that according to Tellier et al. [14], any SETMAR variant with an intact MAR domain can potentially bind centromeres via CENP-B-like sequences, which resemble TIRs. This interpretation was later questioned by Antoine-Lorquin et al. [10], who suggested that the apparent co-localization might largely reflect FL-SETMAR overexpression and could therefore be a methodological artifact. Notably, in their study, no binding of short SETMAR variants to CENP-B-like sequences was detected. Supporting this, a study in haploid cells [16] showed that the only detectable binding sites for FL-SETMAR are *Hsmar1* TIRs, reinforcing the idea that centromeric CENP-B-like sequences are unlikely to be primary targets. Moreover, Tellier et al. [14] proposed that the apparent binding of FL-SETMAR to CENP-B-like sequences may result from its histone methyltransferase activity rather than direct DNA interaction. In this model, FL-SETMAR contributes to establishing or maintaining specific chromatin states in centromeric regions, thereby indirectly associating with CENP-B-defined sequences within a specialized epigenetic environment. Since S-SETMAR lacks the SET domain present in FL-SETMAR, we infer that, even if S-SETMAR overexpression alters the expression of centromeric proteins, its effects at the centromere are likely indirect and not due to direct binding to CENP-B-like sequences. Thus, we assume that the aneuploidy observed following αS-SETMAR overexpression is more likely due to deregulation of genes encoding proteins involved in spindle organization and chromosome segregation, rather than a direct effect of αS-SETMAR at the centromeres.

#### 3.5.3. Apoptosis-Related Genes and Increased Sensitivity to Apoptotic Stimuli

RNA-seq data indicated that apoptosis was moderately affected by αS-SETMAR over-expression, since only 13 genes were concerned (Table S5), with six up-regulated (including PUMA) and seven down-regulated. These results were in line with those previously published, apoptosis having never been identified as a main target for FL-SETMAR. For four up-regulated genes (including *PUMA*), the effect can be attributed to an increase in the number of copies of the gene in αS-SETMAR-8MGBA cells, rather than to a transcriptional effect of αS-SETMAR. Finally, only nine genes involved in apoptosis appeared to be deregulated by αS-SETMAR overexpression, probably accounting for the increased response of αS-SETMAR-8MGBA cells to doxorubicin treatment.

#### 3.5.4. Do *Hsmar1* TIRs Contribute to αS-SETMAR-Dependent Gene Regulation?

All molecular studies of SETMAR raise the question of the role of the *Hsmar1* TIR network. The genomic impact of SETMAR variants has been investigated using ChIP-seq and RNA-seq approaches to identify FL-SETMAR binding sites and transcriptomic alterations. However, the studies published to date [10,14,15,16] relied on distinct cell lines and experimental designs, limiting direct comparisons. Collectively, they indicate that FL-SETMAR preferentially binds *Hsmar1* TIRs or TIR-like sequences and modulates the expression of genes involved in cell cycle control, neuronal functions, and alternative splicing. We therefore asked whether the transcriptional control exerted by αS-SETMAR might occur through its binding to Hsmar1 TIRs. For this purpose, we intersected the gene list given by Tellier et al. [14], which contained genes with Hsmar1 TIRs retaining at least 80% of the length and identity of the ancestral one, with our list of αS-SETMAR deregulated genes. We found that 2.4% (61) of up-regulated genes contained an *Hsmar1* TIR, compared to 4% (103) down-regulated ones (Figure 7C). In addition, we compared the 2571 αS-SETMAR DEGs with those for which an efficient DNA binding has been proven by ChIP-seq analysis. By combining the four studies already published about SETMAR [10,14,16,17], we obtained a list of 214 genes, 18 of which are also deregulated by αS-SETMAR (Figure 7D; Table S2C). These genes were either transcription factors (five genes), involved in cell cycle regulation (four genes) or involved in neurogenesis regulation (six genes). Among them, 12 (eight down- and four up-DEGs) contained an *Hsmar1* TIR. Taken together, we identified 170 genes (164 with a TIR and six without) for which a direct transcriptional effect could be proposed through the binding of αS-SETMAR homodimers in place of FL-SETMAR homodimers. However, these 170 genes represented only 6.6% of all DEGs, suggesting other regulatory mechanisms, via indirect transcriptional effects or alternative pathways, such as the trapping of FL-SETMAR-associated proteins, or αS-SETMAR unknown specific effects.

We then assessed whether some genes can indeed be deregulated following the binding of αS-SETMAR to their sequence. The only study [14] that provided crossed data between ChIP and RNA-seq allowed FL-SETMAR target genes to be identified for which DNA binding was correlated with changes in transcription levels. Five of them were also deregulated by αS-SETMAR (Table S2C, column F). Four were deregulated in the same way—*ABTB1*, *PKHD1* and *SYCP2* (up-regulated), and *BDKRB1* (down-regulated)—and the fifth in the opposite way—*CACNA1A* (down-regulated in our data and up-regulated in Tellier’s data). Interestingly, of the five genes, *CACNA1A* was the only one to be brain-specific, which may explain the divergence in regulation between the two studies, our data having been obtained in CNS cells and those of Tellier in U2OS cells (human osteosarcoma).

Nevertheless, none of the five genes of interest previously identified (*ANXA1*, *HYAL1*, *PLK2*, *SUSD2*, and *RGCC*) was found to be bound by SETMAR in the ChIP-seq list, nor to contain a TIR in their sequence, ruling out a possible direct effect. Instead, indirect effects must be assumed.

*TTN* merited further examination, as its sequence contains an *Hsmar1* TIR. TITINS are giant elastic proteins, the largest known in humans, for which a nuclear isoform has been described. Nuclear TTN associates with chromosomes and is essential for mitotic chromosome condensation and segregation. Studies in *Drosophila* implicate TTN in the organization, stability, elasticity and mechanics of both the nuclear envelope and chromosomes during interphase and mitosis [39]. The down-regulation of TTN in αS-SETMAR-8MGBA cells (LogFC = −1.39) is consistent with the slowdown of the cell cycle associated with αS-SETMAR overexpression. However, it was not identified among the SETMAR-bound loci in the ChIP-seq dataset. Although the possibility of direct αS-SETMAR binding cannot be excluded, it remains to be demonstrated.

Finally, repeated sequences have often been proposed as potential hotspots for chromosomal rearrangements. Notably, no correlation could be established between the chromosomal rearrangement regions detected by aCGH and the *Hsmar1* TIRs. Based on the karyotypic alterations observed in αS-SETMAR-8MGBA cells, we hypothesize that these cells underwent chromoanagenesis during transformation induced by αS-SETMAR, involving a combination of distinct chromosomal events. The precise role of αS-SETMAR and its deregulated target genes (such as *PLK2*, *TTN*, and *RGGC*) remains to be elucidated, opening new avenues for understanding the underlying mechanisms.

## 4. Conclusions

The aim of this work was to understand how αS-SETMAR may exert a protective role in glioblastoma. Our data reveal that expression of this short isoform progressively reshapes proliferative dynamics, slowing overall cell cycle progression and extending cycle duration by approximately 37% (from 27 to 37 h). Rather than reflecting a blockade at a specific checkpoint, this delay appears to arise from a distributed perturbation of cell cycle coordination, consistent with a global loss of temporal fidelity across multiple phases.

At the cytometric level, this deregulation is accompanied by a modest but reproducible accumulation of αS-SETMAR over-expressing cells in G1, together with a striking loss of a sharp G2/M population. Instead, cells in this compartment display a broadened and poorly defined distribution, indicative of impaired synchrony during mitotic progression. This desynchronization is further supported by the presence of abnormal spindle architectures, including tripolar spindles, suggesting defects in spindle assembly and chromosome segregation fidelity. In this context, the slight increase in G1-phase cells likely reflects the propagation of segregation errors, generating heterogeneous daughter populations following defective mitosis.

Functionally, this mitotic instability translates into increased cellular vulnerability. Indeed, αS-SETMAR-expressing cells show reduced survival under stress conditions mimicking therapeutic challenges, consistent with a sensitization to genotoxic stress. This phenotype provides a plausible cellular basis for the favorable prognosis observed in glioblastoma patients exhibiting elevated S-SETMAR levels in peritumoral tissue.

Beyond proliferation defects, these alterations reveal a deeper disruption of genomic integrity. αS-SETMAR expression is associated with profound chromosomal instability, affecting both numerical and structural genome organization. Together, these findings converge on a model in which αS-SETMAR compromises mitotic fidelity, likely through deregulation of spindle and centrosome-associated pathways, thereby linking altered cell cycle dynamics to genomic fragility and therapeutic vulnerability.

The centrosome is an important organizing center in the cell. One of its major roles is to orchestrate the assembly of the mitotic spindle, by ensuring nucleation of microtubules throughout the cell cycle, regulating microtubules organization and assembly within the mitotic spindle. Most cancer cells, in which the occurrence of an abnormal number of centrosomes is common, can group them together to ensure a bipolar spindle. This centrosome clustering takes place shortly before sister chromatid segregation and can lead to aneuploidy, because a cell with three centrosomes will generate a transient tripolar spindle during which the centrosomes attach the kinetochores of the chromosomes via the microtubules. After clustering, the same kinetochore may be connected to two centrosomes at opposite poles, a pattern known as merotelic attachment. If not detected by the SAC, segregation becomes random and can lead to delays in sister chromatid segregation. This is what we observe with the αS-SETMAR-8MGBA lineage, but never in the control line. Merotelic attachments are known to be a major source of aneuploidy in mammalian cells [40]. But in fact, little is known about the mechanisms that drive such catastrophic events. Relationships between aneuploidy and chromosomal disorders can be seen as a vicious cycle, where one potentiates the other.

At this stage, it remains unclear whether aneuploidy preceded chromosomal chaos, occurred concomitantly, or resulted from it. However, a consistent feature of all newly established αS-SETMAR-8MGBA lineages was the induction of these genomic alterations by αS-SETMAR. We propose that, beyond a critical threshold of nuclear disorganization, mitotic progression becomes impaired, triggering a mitotic crisis characterized by tripolar spindle formation and ultimately leading to cell death. Whether this effect is direct or indirect remains unresolved. Although direct binding of αS-SETMAR to the TTN locus may account for the reduced TTN protein levels, it cannot explain the broader transcriptional deregulation observed. Moreover, the lack of *Hsmar1* TIR sequences in most DEGs (≈94%), together with the fact that most of these genes have not been identified as SETMAR binding targets, strongly supports an indirect mechanism, the nature of which remains to be elucidated. Nevertheless, the contribution of *Hsmar1* TIRs cannot be excluded: overexpression of αS-SETMAR in CHO cells, which lack these sequences, did not reveal comparable alterations in proliferation or mitotic defects under our experimental conditions. This observation, however, remains preliminary and was not investigated further.

Beyond its association with chromosomal chaos in the context of αS-SETMAR overexpression, our work further supports a major role for SETMAR in central nervous system development. Our results argue especially in favor of the essential role of S-SETMAR, particularly in synapse biogenesis. Indeed, we have previously shown that S-SETMAR is the predominant variant in stem cells [9] and now that S-SETMAR (but not FL-SETMAR) is bound in a significantly enriched manner to genes involved in neurogenesis and synaptogenesis, especially in glutamatergic synapses.

Finally, the two conclusions highlighted here may appear contradictory: how can a protein (S-SETMAR) involved in brain development be a factor in chromosomal chaos? We propose that this reflects S-SETMAR dosage, or the FL/S-SETMAR ratio. During embryonic development, a certain amount of S-SETMAR is necessary at a specific time for proper neurogenesis. In adult cells, FL-SETMAR (predominant) plays a different role, acting as a guardian of the genome. If, in specific contexts such as oncogenesis, S-SETMAR levels increase again [3], chromosomal chaos can occur. The preferential production of αS-SETMAR, a more stable form of the protein, reinforces this process in GB cells.

These findings raise the possibility of exploiting αS-SETMAR as a therapeutic protein. In practice, this could involve delivering the protein or its coding mRNA/DNA to residual tumor cells in the post-resection cavity, potentially using viral or non-viral vectors, and possibly aided by physical delivery methods such as ultrasound-mediated enhancement. We acknowledge that achieving effective and safe delivery represents a major challenge, and that all cells in the treated area would likely be exposed. Nonetheless, such approaches could impair the remaining tumor cells, including those responsible for recurrence, and improve overall patient prognosis. The response of GB stem cells to αS-SETMAR overexpression is currently under investigation. In addition, S-SETMAR may serve as a prognostic biomarker. In a previous study, we observed that patients with higher S-SETMAR levels in peritumoral tissue tended to be long-term survivors, possibly reflecting a genetic and physiological background more responsive to chemotherapy and radiotherapy. Measuring S-SETMAR levels in surrounding healthy tissue could therefore help define thresholds associated with better prognosis, providing valuable guidance for clinical management.

## Data Availability

Sequencing data have been deposited in GEO under accession number GSE255745. Time-lapse motions of both cell lines are available by clicking on the following link: https://osf.io/j84kd/?view_only=0df478d991db41a6b1c855a90e5cd0e7 (accessed on 17 October 2024).

## Supplementary Materials

The following supporting information can be downloaded at: https://www.mdpi.com/article/10.3390/cancers18132151/s1, Figure S1: plasmid map; Figure S2: 8MGBA cells stably expressing αS-SETMAR; Figure S3: detection of αS-SETMAR/FL-SETMAR heterodimers by IP; Figure S4: time lapse motions; Figure S5: limitations of DNA content-based cell cycle quantification in αS-SETMAR-expressing cells; Figure S6: representative chromosome-level aCGH profiles showing that deregulated gens of interest are not affected by copy number variations; Table S1: list of genes used in the study; Table S2: cross analyses of ChIP-seq and RNA-seq data; Table S3: αS-SETMAR DEGs and cell cycle; Table S4: αS-SETMAR DEGs, spindle and/or chromosomes segregation; Table S5: αS-SETMAR DEGs and apoptosis.
