# Supplementary material for: αS-SETMAR: Inducing Protective Chaos in Glioblastoma?
Source: Cancers (Basel). 2026 Jul 3;18(13):2151. doi: 10.3390/cancers18132151 (PMC13359777; doi:10.3390/cancers18132151)
Supplement: Supplementary file 1 [file cancers-18-02151-s001.zip › David SuppData Rev1.pdf]

αS-SETMAR: inducing a protective chaos in glioblastoma?

Supporting information

Sarah-Anne David, Sara Benharrat, Oriane Lié, Ambre Dufresne, Jérôme Jaillet, Murielle Genty, Sylvaine Renault, Corinne Augé-Gouillou.

Figure S1. Plasmid map

pEF1-V5-αS-SETMAR (7371 bp)

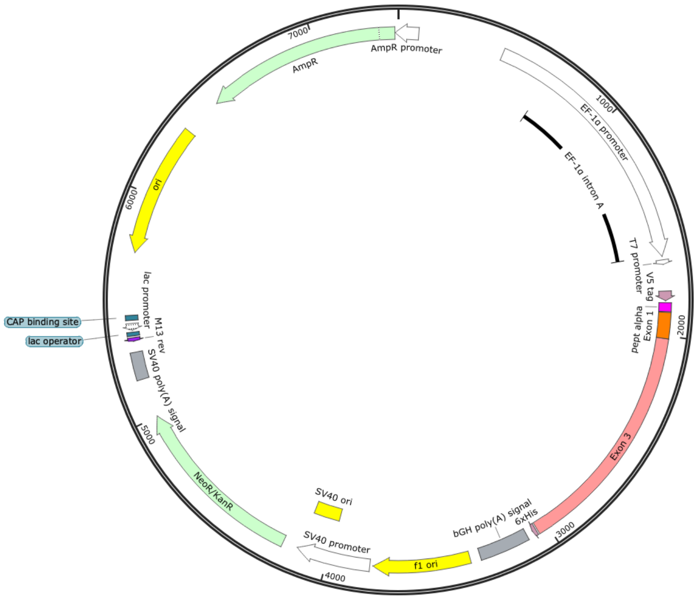

In this construct, the only ATG allowing translation is at the beginning of V5 tag. ATG1 and ATG2, the two alternative ATG driving SETMAR translation, have been mutated.

Consequently, the only translated protein is V5-αS-SETMAR.

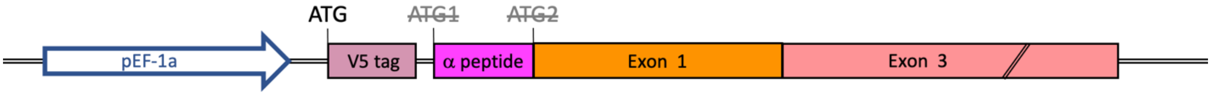

Figure S2. 8MGBA cells stably expressing αS-SETMAR

Four αS-SETMAR-8MGBA clones have been analyzed, and the best one (#1) retained for further work.

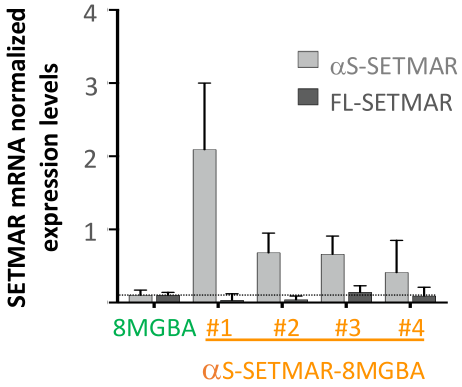

A. RT qPCR analysis of four αS-SETMAR-8MGBA clones (in orange) producing αS-SETMAR, compared to control 8MGBA cells (N=3).

For RT-qPCR analysis, total RNAs were extracted from 8MGBA or αS-SETMAR-8MGBA cells with the Nucleo spin RNA kit (Macherey Nagel) following the supplier's instructions. 11.5 nanograms of total RNA were used for RT reactions (PrimeScript-TM RT Reagent kit, Takara). qPCR were performed with Takyon No ROX SYBR Mastermix blue dTTP (Eurogentec) and specific primers as previously described [11]. qPCR reactions were performed using a Light Cycler 480 II (Roche). cDNA samples were assayed in triplicates for mRNAs expression but in duplicate for mRNAs corresponding to *GAPDH* housekeeping gene. Data were normalized to GAPDH as reference using the 2-ΔΔCp method for analysis.

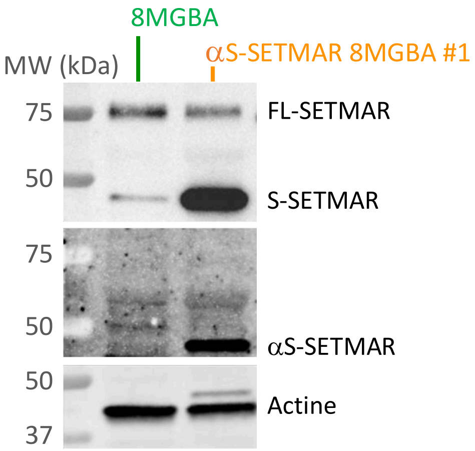

B. Western blot analysis of αS-SETMAR-8MGBA #1, compared to 8MGBA control cells, revealed with: SETMAR antibodies (top panel), αS-SETMAR antibodies (middle panel) and actine antibodies (bottom panel). Molecular weights are indicated in the left margin.

Figure S3. Detection of αS-SETMAR/FL-SETMAR heterodimers by IP

The FL-SETMAR cDNA was cloned downstream of the pEF1 promoter of the pEF1-V5-HisA plasmid (Invitrogen, V92020) giving pEF1-FL-SETMAR, as was done for αS-SETMAR (S1). A FLAG tag was added in 5' of the FL-SETMAR coding sequence and a HA tag in that of αS-SETMAR by *in vitro* mutagenesis using Q5 site-directed mutagenesis kit (New Englands Biolabs).

CHO cells (non-primate, *i.e*. that do not contain endogenous SETMAR) were co-transfected with 0.5 μg of pEIF-HA-αS-SETMAR and 2 μg of pEIF-FLAG-FL-SETMAR with JetOptimus (Polyplus) according to the manufacturer. Culture medium was replaced 7 h after transfection. 48 h after transfection, proteins were extracted in 500 μl of RIPA buffer (Pierce) and 1 X Protease inhibitor cocktail (Pierce), centrifugated for 10 minutes at 12000 g. Proteins were incubated overnight with 2 μg HA antibody (abcam 236632) or FLAG antibody (abcam 205606) cross-linked to 20 μL magnetic beads (ademtech). After washing with RIPA buffer, IP complexes were eluted in 50 mM Glycine, 0.65 % Tween, pH 2.7 and revealed by western blotting.

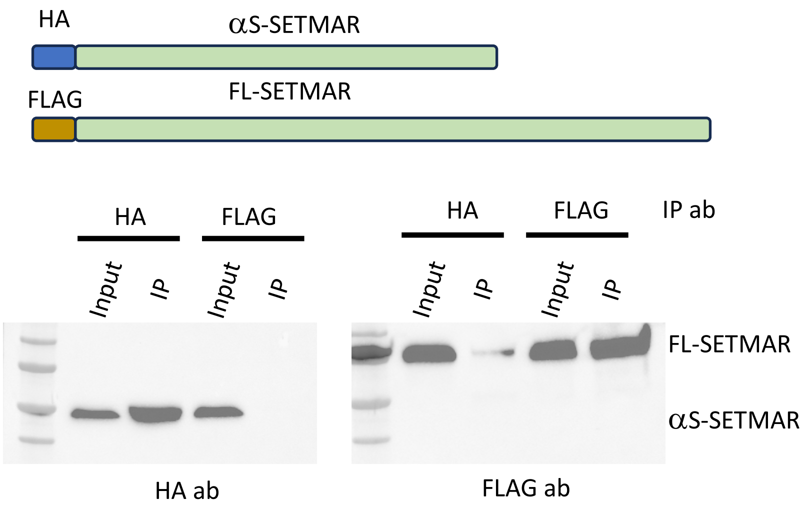

HA antibody allowed the immunoprecipitation of HA-αS-SETMAR (left panel, second line) but also FLAG-FL-SETMAR even if in low quantity (right panel, second line). In contrast, FLAG antibody allowed the immunoprecipitation of FL-SETMAR (right panel, fourth line) but HA-αS-SETMAR did not immunoprecipitated or was not detected because of too low amount (left panel, fourth line).

In conclusion, FL-SETMAR/αS-SETMAR heterodimers does existed, but in low amount or with weak interactions between both proteins.

Figure S4. Time lapse motions

Time lapse motions of both cell lines are available by clicking on the following link:
https://osf.io/j84kd/?view_only=0df478d991db41a6b1c855a90e5cd0e7

Figure S5. Limitations of DNA content–based cell cycle quantification in αS-SETMAR–expressing cells

Due to marked alterations in DNA content profiles, standard automated cell cycle modeling and predefined gating strategies could not be reliably applied. These approaches rely on the presence of discrete, well-resolved DNA content distributions corresponding to canonical cell cycle phases, which are only partially met in 8MGBA cells and clearly disrupted in αS-SETMAR cells.

Indeed, although control 8MGBA-cells display relatively stable DNA content distributions with identifiable G1-, S-, and G2/M-like populations despite underlying aneuploidy, αS-SETMAR–expressing cells exhibit highly heterogeneous and dynamically altered profiles, including loss of a well-defined G2/M peak, expansion of sub-G1 population, and broadening of S-phase.

Consequently, cell cycle distribution analyses were performed using manual gating based on DNA content profiles, enabling comparative analysis across conditions while accounting for the altered distribution of cell populations. This approach introduces some variability in absolute phase estimates, particularly in αS-SETMAR–expressing cells where phase boundaries are poorly defined. Nevertheless, the overall **qualitative** differences between conditions were consistently observed across independent analyses.

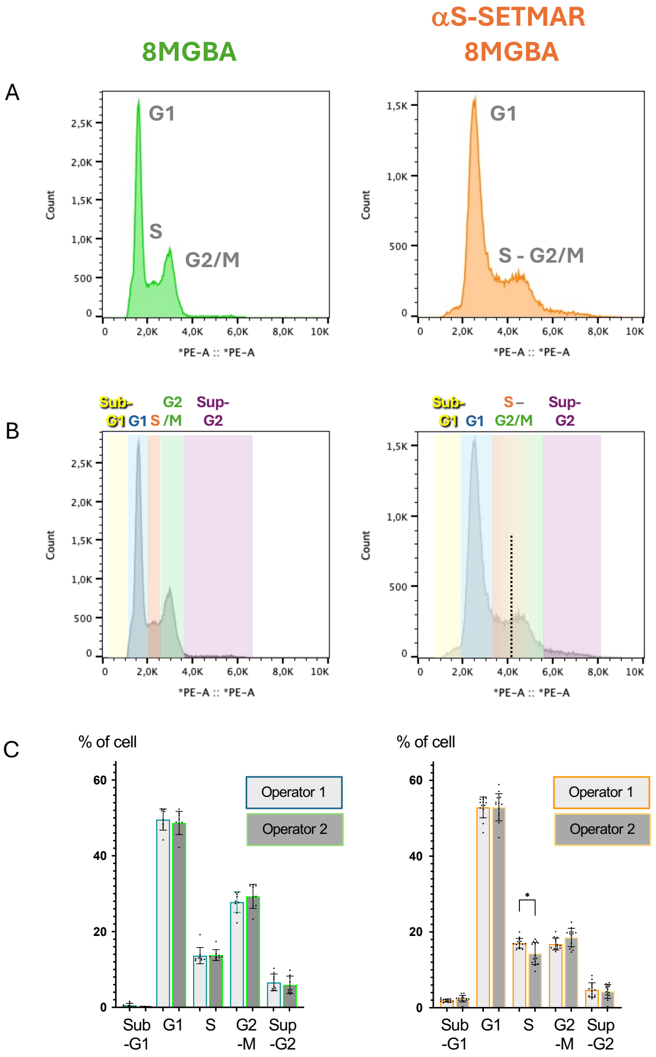

(A) Representative DNA content histograms of control and αS-SETMAR–expressing cells. Control cells display canonical cell cycle profiles with well-resolved G1, S, and G2/M populations. In contrast, αS-SETMAR cells exhibit a marked alteration of DNA content distribution, including broadening of S-phase, loss of a defined G2/M peak, and emergence of a sub-G1 population.

(B) Manual gating strategy. Due to aneuploid DNA content in both conditions (stable in control cells and dynamically altered in αS-SETMAR cells), neither population could be accurately fitted using standard cell cycle deconvolution models. In αS-SETMAR–expressing cells, S-, G2-, and M-phase populations are not clearly separable and form a continuum of DNA content (represented as a color gradient). To enable approximate phase quantification, the right-side foot of the G2/M peak (corresponding to the onset of supra-G2 DNA content) was first identified. Assuming a G2/M peak width comparable to that of the well-resolved G1 population, the lower G2/M boundary (dashed line) was then positioned accordingly within the continuous S-G2/M DNA content distribution.

(C) Phase distribution estimates obtained by independent operator analyses. While control cells show highly reproducible phase distributions, αS-SETMAR–expressing cells display operator-dependent variability mainly in S-phase quantification, reflecting the intrinsic ambiguity in defining boundaries within the continuous S-G2/M DNA content distribution.

In control cells (8MGBA), DNA content profiles allow robust and reproducible phase assignment. In contrast, the heterogeneous and dynamically altered profiles observed in αS-SETMAR–expressing cells preclude reliable quantitative interpretation of phase distributions. As illustrated here, no significant inter-operator differences were observed for phase quantification in 8MGBA cells, whereas a slight but significant difference ($p = 0.048$) was detected for S-phase quantification in αS-SETMAR–expressing cells (17% versus 14%). Consequently, depending on the operator, the apparent difference in S-phase proportion between the two cell lines may appear either marked or limited. These observations therefore led us to restrict our interpretation to qualitative rather than quantitative variations.

Figure S6. Representative chromosome-level aCGH profiles showing that deregulated genes of interest are not affected by copy number variations

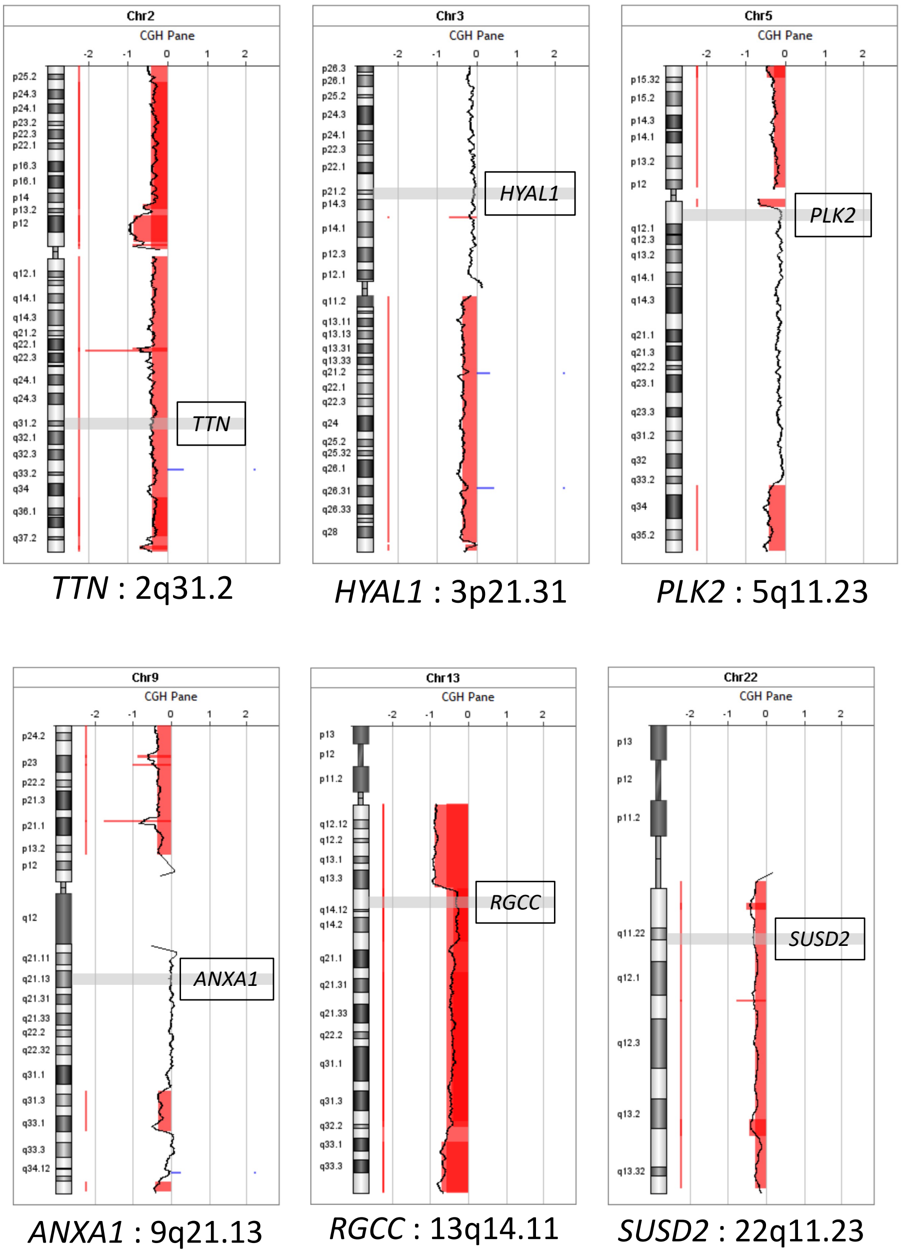

Detailed aCGH profiles are shown for chromosomes carrying deregulated genes of interest (ANXA1, HYAL1, PLK2, SUSD2, RGCC and TTN). Although widespread chromosomal rearrangements and copy number variations are observed at the chromosome level, probe- and segment-level aCGH analyses indicate no copy number alteration at the loci of these genes. Due to the resolution and segmentation of chromosome-level aCGH plots, copy number neutrality at individual gene loci may not always appear as a log2 ratio exactly equal to zero in the graphical representation, as this is the case for *TTN*, *RGCC* and *SUSD2*.
