# Supplementary material for: αS-SETMAR: Inducing Protective Chaos in Glioblastoma?
Source: Cancers (Basel). 2026 Jul 3;18(13):2151. doi: 10.3390/cancers18132151 (PMC13359777; doi:10.3390/cancers18132151)
Supplement: Supplementary file 1 [file cancers-18-02151-s001.zip › original uncropped Western blot.pdf]

Raw blots used for Figure S2

IB : anti-SETMAR

150
100
75
50
37

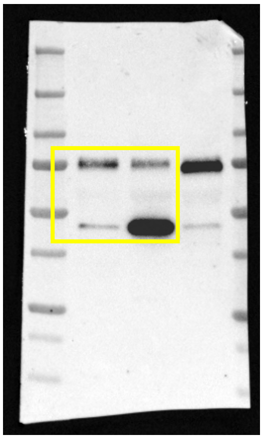

100: 79

Membrane 1

IB : anti-α-peptide

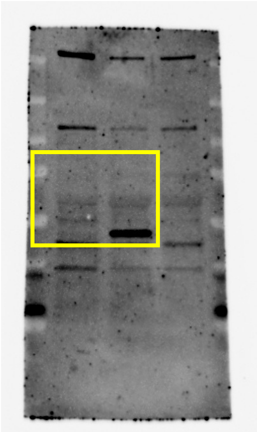

100: 86

Membrane 2

IB : anti-actine

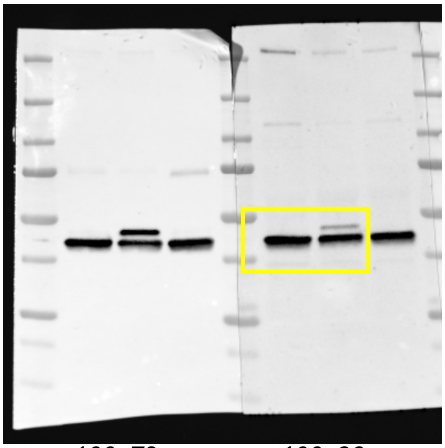

100: 79 *100: 86*

Membrane 1 Membrane 2

Part of the blots used for publication are surrounded by a yellow line

Raw blots used for Figure S3

IB : anti-HA

150
100
75
50
37

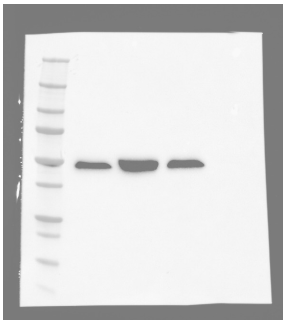

IB : anti-FLAG

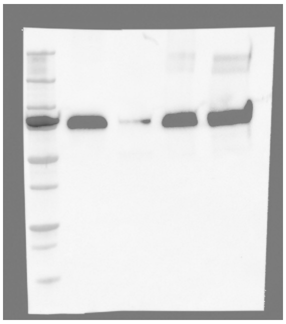

For immunoprecipitation experiments, densitometric lane normalization was not performed because no internal loading control can be applied to IP eluates. Equal volumes corresponding to the entire eluates (30 μl) were loaded in each lane.

Raw blots used for Figure 6

IB : anti-PARPc

150
100
75
50
37

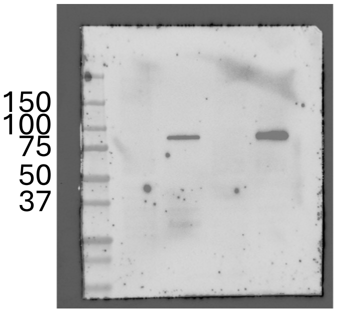

100: 105: 96: 102

IB : anti-actine

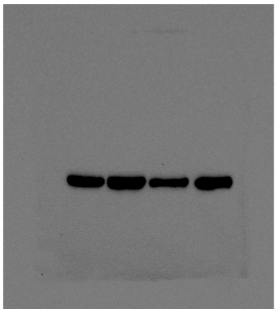

100: 105: 96: 102
